# Ammonium imidazolium dichromate

**DOI:** 10.1107/S1600536812009506

**Published:** 2012-03-10

**Authors:** Run-Qiang Zhu

**Affiliations:** a Ordered Matter Science Research Center, College of Chemistry and Chemical Engineering, Southeast University, Nanjing 211189, People’s Republic of China

## Abstract

In the crystal structure of the title compound, (C_3_H_5_N_2_)(NH_4_)[Cr_2_O_7_], the anions and cations are linked through N—H⋯O hydrogen bonds, resulting in a three-dimensional structure which contains three kinds of layers parallel to (001). One layer contains imidazole cations, the other two layers the ammonium cations and dichromate anions. The dichromate anion has an eclipsed conformation with a dihedral angle of 14.65 (18)° between the mean planes of the O—P—O—P—O backbone.

## Related literature

The title compound was synthesized as part of a search for ferroelectric materials. For general background to ferroelectric compounds with metal-organic framework structures, see: Fu *et al.* (2009 ▶); Ye *et al.* (2006 ▶); Zhang *et al.* (2008 ▶, 2010 ▶). For graph-set motifs, see: Bernstein *et al.* (1995 ▶).
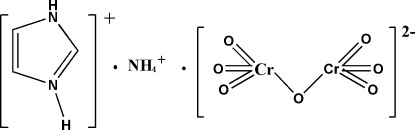


## Experimental

### 

#### Crystal data

(C_3_H_5_N_2_)(NH_4_)[Cr_2_O_7_]
*M* *_r_* = 303.13
Monoclinic, 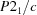

*a* = 5.6260 (11) Å
*b* = 8.2749 (17) Å
*c* = 21.593 (4) Å
β = 91.90 (3)°
*V* = 1004.7 (3) Å^3^
*Z* = 4
Mo *K*α radiation
μ = 2.18 mm^−1^
*T* = 293 K
0.32 × 0.27 × 0.22 mm

#### Data collection

Rigaku SCXmini diffractometer
Absorption correction: multi-scan (*CrystalClear*; Rigaku, 2005 ▶) *T*_min_ = 0.502, *T*_max_ = 0.618
10091 measured reflections
2297 independent reflections
1907 reflections with *I* > 2σ(*I*)
*R*_int_ = 0.047

#### Refinement

*R*[*F* ^2^ > 2σ(*F* ^2^)] = 0.037
*wR*(*F* ^2^) = 0.085
*S* = 1.09
2297 reflections
152 parameters
H atoms treated by a mixture of independent and constrained refinement
Δρ_max_ = 0.34 e Å^−3^
Δρ_min_ = −0.52 e Å^−3^

### 

Data collection: *CrystalClear* (Rigaku, 2005 ▶); cell refinement: *CrystalClear*; data reduction: *CrystalClear*; program(s) used to solve structure: *SHELXS97* (Sheldrick, 2008 ▶); program(s) used to refine structure: *SHELXL97* (Sheldrick, 2008 ▶); molecular graphics: *DIAMOND* (Brandenburg & Putz, 2005 ▶); software used to prepare material for publication: *SHELXL97*.

## Supplementary Material

Crystal structure: contains datablock(s) I, global. DOI: 10.1107/S1600536812009506/fj2526sup1.cif

Structure factors: contains datablock(s) I. DOI: 10.1107/S1600536812009506/fj2526Isup2.hkl

Additional supplementary materials:  crystallographic information; 3D view; checkCIF report

## Figures and Tables

**Table 1:** Hydrogen-bond geometry (Å, °)

*D*—H⋯*A*	*D*—H	H⋯*A*	*D*⋯*A*	*D*—H⋯*A*
N2—H2*B*⋯O7^i^	0.86	2.16	3.011 (4)	170
N3—H3*B*⋯O1^ii^	0.86	2.04	2.827 (4)	152
N1—H1*B*⋯O7	0.79 (5)	2.16 (5)	2.940 (4)	169 (5)
N1—H1*C*⋯O4^iii^	0.83 (5)	2.19 (5)	2.943 (4)	151 (5)
N1—H1*D*⋯O3^iv^	0.71 (5)	2.29 (5)	3.004 (5)	176 (5)
N1—H1*E*⋯O6^v^	0.74 (5)	2.15 (5)	2.895 (4)	174 (5)

Symmetry codes: (i) 
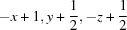
; (ii) 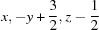; (iii) 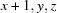; (iv) 
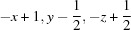
; (v) 
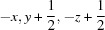
.

**Fig. 1.**
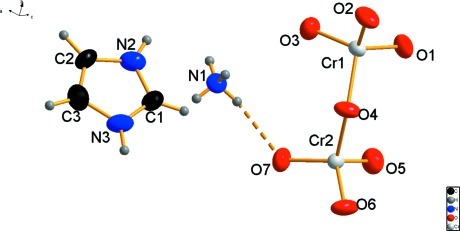
The molecular structure of the title compound, with the displacement ellipsoids drawn at the 30% probability level.

**Fig. 2.**
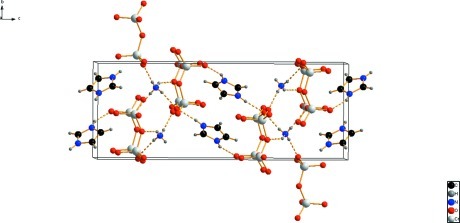
Packing diagram of the title compound, showing the structure along the a axis. Hydrogen bonds are shown as dashed lines.

## supplementary crystallographic information

### Comment

We synthesized the title compound to find ferroelectric material by dielectric measurements of compound as a function of temperature(Fu *et al.*, 2009; Ye *et al.*, 2006; Zhang *et al.*, 2008; Zhang *et al.*, 2010). In the range from 190 K to near its melting point (m.p. >370 K), no dielectric anomaly was observed.

A view of the title compound is shown in Fig.1. The structure is consolidated by multiple intermolecular and intramolecular hydrogen bonds between N and O. This hydrogen bondings (table 1, Fig.2) produces a three-dimensional net work. The N···O distances of the hydrogen bonding are in the range of 2.827 (4) – 3.011 (4) for table 1. Hydrogen bonding is the most reliable desigen element in the non-covalent assembly of molecules with donor and accept functionalities, and as such it is the most important interaction in crystal engineering (Bernstein *et al.*, 1995).

### Experimental

A mixture of imidazole (0.68 g, 10 mmol), ammonium dichromate (2.5 g, 10 mmol) in water was stirred for several days at ambient temperature, red sheet crystals were obtained.

### Refinement

Hydrogen atom positions were calculated and allowed to ride on their parent atoms with aromtic C–H = 0.93 Å and N–H = 0.86 Å, and with *U*ĩso(H)=1.2*U*eq(C or N).The H atoms on N1 were freely refined.

### Crystal data

**Table:**

(C_3_H_5_N_2_)(NH_4_)[Cr_2_O_7_]	*F*(000) = 608
*M**_r_* = 303.13	*D*_x_ = 2.004 Mg m^−^^3^
Monoclinic, *P*2_1_/*c*	Mo *K*α radiation, λ = 0.71073 Å
Hall symbol: -P 2ybc	Cell parameters from 2297 reflections
*a* = 5.6260 (11) Å	θ = 3.1–27.5°
*b* = 8.2749 (17) Å	µ = 2.18 mm^−^^1^
*c* = 21.593 (4) Å	*T* = 293 K
β = 91.90 (3)°	Plane, red
*V* = 1004.7 (3) Å^3^	0.32 × 0.27 × 0.22 mm
*Z* = 4	

### Data collection

**Table:**

Rigaku SCXmini diffractometer	2297 independent reflections
Radiation source: fine-focus sealed tube	1907 reflections with *I* > 2σ(*I*)
Graphite monochromator	*R*_int_ = 0.047
CCD Profile fitting scans	θ_max_ = 27.5°, θ_min_ = 3.1°
Absorption correction: multi-scan (*CrystalClear*; Rigaku, 2005)	*h* = −7→7
*T*_min_ = 0.502, *T*_max_ = 0.618	*k* = −10→10
10091 measured reflections	*l* = −28→27

### Refinement

**Table:**

Refinement on *F*^2^	Primary atom site location: structure-invariant direct methods
Least-squares matrix: full	Secondary atom site location: difference Fourier map
*R*[*F*^2^ > 2σ(*F*^2^)] = 0.037	Hydrogen site location: inferred from neighbouring sites
*wR*(*F*^2^) = 0.085	H atoms treated by a mixture of independent and constrained refinement
*S* = 1.09	*w* = 1/[σ^2^(*F*_o_^2^) + (0.0321*P*)^2^ + 0.944*P*] where *P* = (*F*_o_^2^ + 2*F*_c_^2^)/3
2297 reflections	(Δ/σ)_max_ < 0.001
152 parameters	Δρ_max_ = 0.34 e Å^−^^3^
0 restraints	Δρ_min_ = −0.52 e Å^−^^3^

### Special details

**Table:**

Geometry. All e.s.d.'s (except the e.s.d. in the dihedral angle between two l.s. planes) are estimated using the full covariance matrix. The cell e.s.d.'s are taken into account individually in the estimation of e.s.d.'s in distances, angles and torsion angles; correlations between e.s.d.'s in cell parameters are only used when they are defined by crystal symmetry. An approximate (isotropic) treatment of cell e.s.d.'s is used for estimating e.s.d.'s involving l.s. planes.
Refinement. Refinement of *F*^2^ against ALL reflections. The weighted *R*-factor *wR* and goodness of fit *S* are based on *F*^2^, conventional *R*-factors *R* are based on *F*, with *F* set to zero for negative *F*^2^. The threshold expression of *F*^2^ > σ(*F*^2^) is used only for calculating *R*-factors(gt) *etc*. and is not relevant to the choice of reflections for refinement. *R*-factors based on *F*^2^ are statistically about twice as large as those based on *F*, and *R*- factors based on ALL data will be even larger.

### Fractional atomic coordinates and isotropic or equivalent isotropic displacement parameters (Å^2^)

**Table:**

	*x*	*y*	*z*	*U*_iso_*/*U*_eq_	
C1	0.3469 (6)	0.7554 (4)	0.06526 (17)	0.0396 (8)	
H1A	0.2345	0.7447	0.0956	0.048*	
C2	0.6599 (6)	0.8268 (4)	0.01522 (17)	0.0390 (8)	
H2A	0.8034	0.8757	0.0060	0.047*	
C3	0.5350 (7)	0.7289 (5)	−0.02122 (18)	0.0473 (9)	
H3A	0.5725	0.6965	−0.0610	0.057*	
N1	0.5234 (6)	0.7387 (4)	0.24161 (17)	0.0325 (6)	
N2	0.5405 (5)	0.8437 (4)	0.06922 (14)	0.0437 (7)	
H2B	0.5851	0.9023	0.1004	0.052*	
N3	0.3399 (5)	0.6843 (3)	0.01056 (15)	0.0449 (8)	
H3B	0.2299	0.6200	−0.0030	0.054*	
O1	0.0282 (4)	0.9810 (3)	0.42701 (10)	0.0378 (5)	
O2	−0.0350 (5)	1.1228 (3)	0.31828 (11)	0.0422 (6)	
O3	0.3641 (4)	0.9650 (3)	0.34399 (12)	0.0429 (6)	
O4	−0.0528 (4)	0.7910 (2)	0.32273 (11)	0.0375 (5)	
O5	−0.0001 (5)	0.5720 (3)	0.41884 (11)	0.0458 (6)	
O6	−0.2197 (4)	0.4834 (3)	0.31508 (12)	0.0425 (6)	
O7	0.2496 (4)	0.5232 (3)	0.32071 (11)	0.0415 (6)	
Cr1	0.08035 (8)	0.97044 (5)	0.35383 (2)	0.02452 (14)	
Cr2	−0.00312 (8)	0.58625 (5)	0.34477 (2)	0.02334 (14)	
H1B	0.439 (8)	0.692 (6)	0.264 (2)	0.066 (16)*	
H1C	0.644 (9)	0.783 (6)	0.256 (2)	0.081 (18)*	
H1D	0.556 (8)	0.677 (6)	0.221 (2)	0.051 (15)*	
H1E	0.439 (8)	0.796 (6)	0.226 (2)	0.059 (15)*	

### Atomic displacement parameters (Å^2^)

**Table:**

	*U*^11^	*U*^22^	*U*^33^	*U*^12^	*U*^13^	*U*^23^
C1	0.0366 (18)	0.0382 (19)	0.044 (2)	−0.0071 (15)	0.0056 (15)	0.0040 (16)
C2	0.0287 (16)	0.0377 (18)	0.051 (2)	−0.0066 (14)	0.0055 (15)	0.0081 (16)
C3	0.051 (2)	0.051 (2)	0.040 (2)	0.0087 (18)	0.0061 (17)	−0.0013 (18)
N1	0.0337 (17)	0.0280 (16)	0.0357 (17)	0.0060 (14)	−0.0008 (14)	0.0038 (14)
N2	0.0505 (18)	0.0362 (16)	0.0438 (18)	−0.0065 (14)	−0.0080 (14)	−0.0024 (13)
N3	0.0370 (16)	0.0365 (16)	0.060 (2)	−0.0108 (13)	−0.0108 (14)	−0.0028 (15)
O1	0.0463 (13)	0.0415 (13)	0.0256 (11)	0.0090 (11)	0.0029 (10)	−0.0019 (10)
O2	0.0659 (16)	0.0254 (11)	0.0352 (13)	0.0112 (11)	0.0000 (11)	0.0048 (10)
O3	0.0323 (12)	0.0482 (14)	0.0485 (15)	−0.0032 (11)	0.0072 (11)	−0.0030 (12)
O4	0.0465 (13)	0.0199 (10)	0.0452 (14)	−0.0002 (9)	−0.0130 (11)	0.0003 (9)
O5	0.0538 (15)	0.0505 (15)	0.0334 (13)	0.0077 (12)	0.0042 (11)	0.0057 (11)
O6	0.0324 (12)	0.0254 (11)	0.0686 (17)	−0.0075 (9)	−0.0132 (11)	0.0037 (11)
O7	0.0290 (12)	0.0459 (14)	0.0501 (15)	0.0066 (10)	0.0109 (11)	0.0050 (11)
Cr1	0.0307 (3)	0.0190 (2)	0.0239 (3)	0.00146 (18)	0.00126 (19)	−0.00066 (18)
Cr2	0.0199 (2)	0.0209 (2)	0.0292 (3)	0.00099 (17)	0.00006 (18)	0.00220 (18)

### Geometric parameters (Å, º)

**Table:**

C1—N2	1.312 (4)	N1—H1E	0.74 (5)
C1—N3	1.319 (5)	N2—H2B	0.8600
C1—H1A	0.9300	N3—H3B	0.8600
C2—C3	1.316 (5)	O1—Cr1	1.619 (2)
C2—N2	1.372 (5)	O2—Cr1	1.602 (2)
C2—H2A	0.9300	O3—Cr1	1.618 (2)
C3—N3	1.364 (5)	O4—Cr2	1.779 (2)
C3—H3A	0.9300	O4—Cr1	1.784 (2)
N1—H1B	0.79 (5)	O5—Cr2	1.603 (2)
N1—H1C	0.83 (5)	O6—Cr2	1.602 (2)
N1—H1D	0.71 (5)	O7—Cr2	1.616 (2)
			
N2—C1—N3	107.8 (3)	C2—N2—H2B	125.8
N2—C1—H1A	126.1	C1—N3—C3	109.2 (3)
N3—C1—H1A	126.1	C1—N3—H3B	125.4
C3—C2—N2	107.7 (3)	C3—N3—H3B	125.4
C3—C2—H2A	126.1	Cr2—O4—Cr1	129.15 (13)
N2—C2—H2A	126.1	O2—Cr1—O3	110.17 (13)
C2—C3—N3	106.8 (3)	O2—Cr1—O1	109.98 (12)
C2—C3—H3A	126.6	O3—Cr1—O1	109.94 (13)
N3—C3—H3A	126.6	O2—Cr1—O4	108.44 (11)
H1B—N1—H1C	119 (5)	O3—Cr1—O4	109.34 (12)
H1B—N1—H1D	102 (5)	O1—Cr1—O4	108.94 (12)
H1C—N1—H1D	110 (5)	O6—Cr2—O5	110.06 (14)
H1B—N1—H1E	102 (4)	O6—Cr2—O7	111.42 (13)
H1C—N1—H1E	113 (5)	O5—Cr2—O7	108.47 (13)
H1D—N1—H1E	110 (5)	O6—Cr2—O4	106.74 (11)
C1—N2—C2	108.5 (3)	O5—Cr2—O4	109.46 (12)
C1—N2—H2B	125.8	O7—Cr2—O4	110.69 (12)

### Hydrogen-bond geometry (Å, º)

**Table:**

*D*—H···*A*	*D*—H	H···*A*	*D*···*A*	*D*—H···*A*
N2—H2*B*···O7^i^	0.86	2.16	3.011 (4)	170
N3—H3*B*···O1^ii^	0.86	2.04	2.827 (4)	152
N1—H1*B*···O7	0.79 (5)	2.16 (5)	2.940 (4)	169 (5)
N1—H1*C*···O4^iii^	0.83 (5)	2.19 (5)	2.943 (4)	151 (5)
N1—H1*D*···O3^iv^	0.71 (5)	2.29 (5)	3.004 (5)	176 (5)
N1—H1*E*···O6^v^	0.74 (5)	2.15 (5)	2.895 (4)	174 (5)

Symmetry codes: (i) −*x*+1, *y*+1/2, −*z*+1/2; (ii) *x*, −*y*+3/2, *z*−1/2; (iii) *x*+1, *y*, *z*; (iv) −*x*+1, *y*−1/2, −*z*+1/2; (v) −*x*, *y*+1/2, −*z*+1/2.
